# Patient-Reported Goals of Youths in Canada Receiving Medication-Assisted Treatment for Opioid Use Disorder

**DOI:** 10.1001/jamanetworkopen.2021.19600

**Published:** 2021-08-05

**Authors:** Darren Chai, Tea Rosic, Balpreet Panesar, Nitika Sanger, Emma A. van Reekum, David C. Marsh, Andrew Worster, Lehana Thabane, Zainab Samaan

**Affiliations:** 1 Michael G. DeGroote School of Medicine, McMaster University, Hamilton, Ontario, Canada; 2 Department of Psychiatry and Behavioral Neurosciences, McMaster University, Hamilton, Ontario, Canada; 3 Department of Health Research Methods, Evidence, and Impact, McMaster University, Hamilton, Ontario, Canada; 4 Neurosciences Graduate Program, McMaster University, Hamilton, Ontario, Canada; 5 Medical Sciences Graduate Program, McMaster University, Hamilton, Ontario, Canada; 6 Northern Ontario School of Medicine, Sudbury, Ontario, Canada; 7 Canadian Addiction Treatment Centres, Markham, Ontario, Canada; 8 Institute for Clinical Evaluative Sciences North, Sudbury, Ontario, Canada; 9 Department of Medicine, McMaster University, Hamilton, Ontario, Canada; 10 Biostatistics Unit, Research Institute at St Joseph’s Healthcare, Hamilton, Ontario, Canada; 11 Clinician Investigator Program, Mood Disorders Program, St Joseph’s Healthcare, Hamilton, Ontario, Canada

## Abstract

**Importance:**

In the literature on opioid use disorder (OUD), opioid abstinence is used as an outcome measure for individuals receiving medication-assisted treatment (MAT), without consideration of patient-reported goals (PRGs).

**Objectives:**

To identify common PRGs for youths receiving MAT for OUD and assess whether these patients achieve their stated goals.

**Design, Setting, and Participants:**

This prospective cohort study examined data from 152 individuals aged 16 to 25 years (noninclusive) recruited between May 22, 2018, and March 11, 2020, from 45 outpatient MAT clinics in the Pharmacogenetics of Opioid Substitution Treatment Response study. Youths receiving MAT for OUD were included and were followed up for 3 months.

**Exposures:**

Medication-assisted treatment for OUD.

**Main Outcomes and Measures:**

The frequency of each PRG; the success of goal attainment, compared between those who reported specific PRGs and those who did not; and associations between reporting certain goals and achieving them.

**Results:**

Among the 152 youths in the study, 82 were male (53.9%), and the mean (SD) age was 22.8 (1.8) years. Ten overarching goals were identified, with the most common being to taper the dose of or stop MAT (96 [63.2%]), avoid use of recreational substances (71 [46.7%]), manage OUD symptoms (25 [16.4%]), live a normal life (14 [9.2%]), improve mental health (11 [7.2%]), and gain employment (8 [5.3%]). Overall, individuals who reported PRGs had similar odds of achieving them as those who did not for the goals of taper dose of or stop MAT (OR, 1.98; 95% CI, 0.88-4.46; *P* = .10), avoid recreational substances (OR, 1.34; 95% CI, 0.65-2.74; *P* = .43), manage OUD symptoms (β coefficient, –0.93; 95% CI, –4.24 to 2.38; *P* = .58), and improve mental health (β coefficient, –0.76; 95% CI, –6.31 to 4.78; *P* = .79). Furthermore, multivariable logistic regression showed that goals to taper the dose of or stop MAT (odds ratio, 1.90; 95% CI, 0.78-4.63; *P* = .16) or avoid recreational substances (odds ratio, 1.27; 95% CI, 0.60-2.67; *P* = .53) were not associated with achieving these respective outcomes.

**Conclusions and Relevance:**

This study suggests that youths have highly variable PRGs regarding MAT for OUD and that reporting a goal may not mean one is at higher odds of achieving it. There is a need to develop treatment plans that effectively incorporate PRGs. In addition, the finding that most youths aim to minimize or stop their MAT dose warrants the creation of a tapering protocol to guide clinicians. Because a diagnosis of OUD has substantial psychosocial implications in this population, clinicians must ensure that these dimensions of care are part of routine clinical practice.

## Key Points

**Question:**

What are the self-reported treatment goals of youths receiving medication-assisted treatment (MAT) for opioid use disorder, and are those goals achieved?

**Findings:**

In this cohort study of 152 participants, 63% reported wanting to taper off their MAT dose or stop MAT, 47% reported wanting to avoid recreational substances, and 16% reported wanting to manage their opioid use disorder symptoms. Participants who reported these goals did not have significantly higher odds of achieving them than those who did not.

**Meaning:**

This study suggests that youths have highly variable goals for MAT for opioid use disorder, but having such goals is not associated with increased odds of achieving them.

## Teaser

This cohort study uses data from the Pharmacogenetics of Opioid Substitution Treatment Response study to identify common patient-reported goals for youths receiving medication-assisted treatment for opioid use disorder (OUD) and assesses whether these patients achieve their stated goals.

## Introduction

In the last decade, more emphasis has been placed on understanding patients’ goals in the treatment of substance use disorders.^1,2,3^ The concept of patient-centered care, which was originally defined as “care that is respectful of, and responsive to, the individual patient preferences, needs, and values,”^4,5^^(p780)^ has a positive association with patient outcomes and satisfaction with care.^6^ However, studies have also shown that patients’ preferred treatment goals do not always align with those of the health care system.^7,8,9^ Moreover, this discordance exists despite evidence that patients in treatment for substance use disorders would like to be more involved in their care planning.^10,11^

Despite recent efforts to control Canada’s opioid crisis,^12,13^ there was a 50% increase in total opioid-related deaths between 2016 and 2018.^12^ Compared with the general population, youths (defined in this study as individuals aged 16-25 years, noninclusive) have a disproportionately increased prevalence of recreational opioid use.^14^ In Canada, the highest rates of recreational opioid use are among youths aged 20 to 24 years (1.1%) and those aged 15 to 19 years (0.8%).^14^

Medication-assisted treatment (MAT) refers to the use of prescription medications as part of the treatment for individuals with opioid use disorder (OUD). Currently, the 3 medications approved by the US Food and Drug Administration to treat OUD include methadone hydrochloride, buprenorphine hydrochloride, and naltrexone hydrochloride.^15^ Several studies have examined the effectiveness of MAT for youths with OUD.^16,17,18,19,20,21,22^ Overall, the literature supports the use of MAT in this population, evidenced by the superior abstinence rates and better treatment retention.^23,24^ Moreover, the American Academy of Pediatrics released a policy statement advocating for more resources to improve access to MAT in this population and for further research in this field.^23,24^

There are no studies within the OUD literature, to our knowledge, that evaluate patient-reported goals (PRGs) for youths receiving MAT for OUD. A previous study has shown that patients receiving MAT for OUD have treatment goals that are inconsistent with outcomes often measured in clinical trials and treatment programs.^25^ Although some studies measure changes in psychosocial functioning (eg, school performance, work performance, and family relationships),^16,17^ most studies focus primarily on abstinence and treatment retention.^18,19,20,21,22^ Furthermore, a study has shown that there is no association between achieving opioid abstinence and most of these PRGs.^26^ However, we do not know whether youths who report PRGs have increased odds of achieving their self-reported goals. Therefore, in this study, we aimed to identify PRGs for youths receiving MAT for OUD using a mixed-methods approach and to explore whether youths achieve their goals in treatment.

## Methods

### Data Source

We collected prospective observational data from 152 youths aged 16 to 25 years, noninclusive, undergoing MAT for OUD. These youths were enrolled in the Pharmacogenetics of Opioid Substitution Treatment Response study, which recruited 2030 participants between May 22, 2018, and March 11, 2020, from 45 outpatient MAT clinics in Ontario, Canada. Study methods have been previously published.^25,26^ We used a convenience sampling strategy at a network of MAT clinics in Ontario, Canada, in which patients were consecutively approached for study participation during their regularly scheduled appointments and recruited voluntarily. Participants could be enrolled in MAT for any length of time before study enrollment. They received clinical care per clinic protocol, which included physician follow-up, medication adjustments, and regular urine drug screening tests (UDSs). All participating clinics followed the same protocols and were managed centrally through a single management team. Participants met our inclusion criteria if they were (1) older than 16 years to younger than 25 years, noninclusive, and (2) receiving MAT for a diagnosis of OUD, as per the *Diagnostic and Statistical Manual of Mental Disorders* (Fifth Edition) (*DSM-5*).^27^ Individuals were excluded if they were (1) aged 16 years or younger, (2) aged 25 years or older, or (3) not receiving MAT for a diagnosis of OUD, as per the *DSM-5*^27^ (Figure). This study was approved by and conducted per the ethical guidelines of the Hamilton Integrated Research Ethics Board; all participants provided written informed consent. This study is reported in adherence to the Strengthening the Reporting of Observational Studies in Epidemiology (STROBE) reporting guideline for observational studies.^28^

**Figure.**
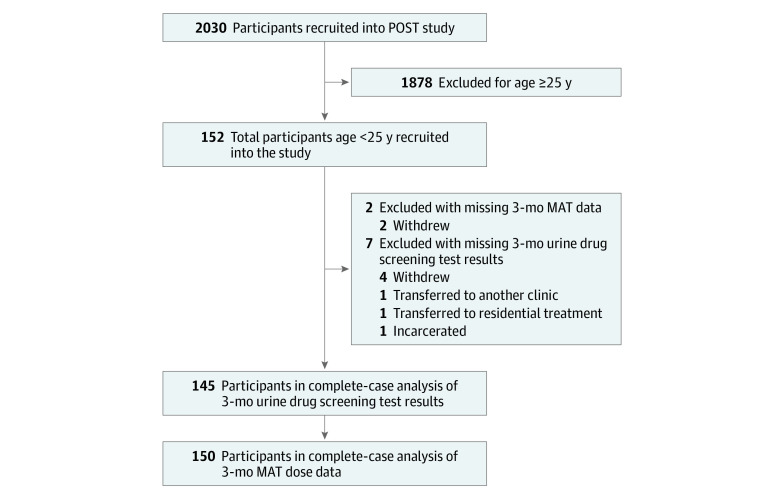
Study Flow Diagram MAT indicates medication-assisted treatment; POST, Pharmacogenetics of Opioid Substitution Treatment Response.

Participants completed face-to-face interviews at study enrollment to collect information on demographic and clinical characteristics. All participants were asked, “What are your goals in treatment?” We used NVivo software, version 12 (QSR International [Americas] Inc) for qualitative analysis to identify common themes from patients’ answers.^29^ We did not limit the number of goals a patient could report. First, the responses to this question were reviewed in Microsoft Excel (Microsoft Corp) to minimize typographic errors and better understand these data. Next, these data were loaded into NVivo to catalog the main ideas, phrases, and patterns into nodes using free-text queries. These queries were performed to capture patterns in these data and improve analytic accuracy by identifying stemmed variants. Finally, regular housekeeping of nodes was performed that included combining related nodes. These steps were repeated several times to allow well-researched nodes to become themes (eTable 1 in the Supplement). Ultimately, we identified 10 distinct treatment goals: taper MAT dose or stop MAT, avoid recreational substances, manage OUD symptoms, live a normal life, improve mental health, employment, maintain or stabilize MAT dose, family, education, and pain management.

Urine drug screening tests were conducted using the FaStep Assay (Trimedic Supply Network Ltd) to detect morphine, oxycodone, fentanyl, methadone metabolites, buprenorphine, cocaine, amphetamines, and methamphetamines.^30^ In this study, we refer to members of the aforementioned list, excluding methadone metabolites and buprenorphine, as “recreational substances.” The data from UDSs, performed for up to 12 months before study enrollment (following clinic protocol, typically weekly or biweekly), were made available and considered “baseline.” Because participants were enrolled in MAT for variable durations, the number of UDSs performed for each participant varies. After enrollment, we followed up with participants for 3 months with continued UDSs. Three months was the chosen follow-up period to retain a reasonable time proximity between when participants stated their PRGs and their final outcome assessments.

### Statistical Analysis

We conducted all quantitative analyses using Stata, version 15.1 (StataCorp LP), with the level of significance for hypothesis testing set at α = .05; all hypothesis testing was 2-sided. Demographic and clinical data were summarized using mean (SD) values if the distributions of continuous variables appeared approximately normal. Skewed data were reported as median values with interquartile ranges. Categorical variables were reported as frequency with percentage.

Abstinence from opioids was defined as no positive UDS results for opioids, other than methadone and buprenorphine, during the period of interest. Because we did not know whether all of the participants had previously used other nonopioid recreational substances, we could not comment on whether they were “abstinent” based on negative results of UDSs or simply “nonusers.” Therefore, we defined other recreational substance nonusers as individuals with no positive UDS results for each respective substance during the period of interest (ie, baseline or 3 months). Both baseline and 3-month UDS data for opioids were summarized in 3 ways: the mean (SD) number of UDSs performed, the number of patients who were opioid abstinent, and the percentage of opioid-positive UDS results among nonabstinent participants. All other data on substance use were summarized in 2 ways: the mean number of UDSs for the respective substance and being a “nonuser” of the respective substance.

Using either univariable logistic regression or univariable linear regression, we examined the success of achieving 5 PRGs for which we had available outcome data to measure. The 5 goals and their respective outcome measures were as follows: taper the dose or stop MAT, measured by the proportion of individuals who decreased their MAT dose during the 3-month study period; avoid recreational substances, measured by the proportion of individuals who had negative UDS results for all recreational substances during the 3-month study period; employment, measured by the proportion of individuals who were employed at enrollment; manage OUD symptoms, measured by the total physical symptom score on the Maudsley Addiction Profile (MAP)^31^ at enrollment; and improve mental health, measured by the total psychological symptom score on the MAP at enrollment. In all 5 regressions, individuals who reported these goals were compared with individuals who did not, and the respective outcome measures were the dependent variables in each respective model. Furthermore, we also used multivariable logistic regression for the 2 most commonly reported goals, taper dose or stop MAT and avoid recreational substances. Again, the dependent variables in these models were the outcome measures for each goal. In both models, we controlled for important covariates believed to be associated with MAT outcomes: age, sex,^32,33^ type of MAT (methadone or buprenorphine-naloxone), medication dose,^34^ and length of time receiving treatment.^35^ Results were reported as either odds ratios (ORs) or β coefficients, both with 95% CIs and associated *P* values. Finally, we assessed for multicollinearity using the variance inflation factor and confirmed that it was less than 2 for all included covariates.

### Missing Data

The number of participants with missing data and the reasons for missingness are reported in the Figure. Of 152 participants, 7 (4.6%) had missing 3-month UDS data, and 2 (1.3%) had missing 3-month MAT dose data. We used complete-case analysis because our outcomes were unlikely to be associated with the low proportion of missing values. A comparison of participants with complete and incomplete data can be found in eTable 2 in the Supplement.

## Results

### Sample

This study included data for 152 youths (82 male participants [53.9%] and 70 female participants [46.1%]) aged 16.7 to 24.9 years (mean [SD] age, 22.8 [1.8] years) (Table 1). At baseline, 106 participants (69.7%) were receiving methadone, while the remaining 46 (30.3%) received buprenorphine. The median length of treatment was 0.8 years (interquartile range, 0.2-2.0 years).

**Table 1.** Demographic and Clinical Characteristics of 152 Youths

Characteristic	Youths, No. (%)
Age, y	
Mean (SD)	22.8 (1.8)
Range	16.7-24.9
Sex	
Female	70 (46.1)
Male	82 (53.9)
Socioeconomic factors	
Patients with children	56 (36.8)
Currently employed	45 (29.6)
Level of education	
<High school	70 (46.1)
High school completed	73 (48.0)
Postsecondary education in progress or completed	9 (5.9)
MAT history	
Type of MAT	
Methadone	106 (69.7)
Buprenorphine	46 (30.3)
Baseline dose, mean (SD) mg/d	
Methadone	58.7 (33.2)
Buprenorphine	11.0 (6.7)
Length of treatment, median (IQR), y	0.8 (0.2-2.0)
Substance use history^a^	
Participants who were opioid abstinent	
At baseline	52 (34.2)
At 3 mo^b^	78 (53.8)
Any recreational substance use	
At baseline	126 (82.9)
At 3 mo^b^	102 (70.3)
Cocaine nonuser^c^	
At baseline	62 (40.8)
At 3 mo^b^	76 (52.4)
Amphetamine nonuser^c^	
At baseline	94 (61.8)
At 3 mo^b^	102 (70.3)
Methamphetamine nonuser^c^	
At baseline	112 (73.7)
At 3 mo^b^	115 (79.3)
Patient-reported goals in treatment^d^	
Taper dose or stop MAT	96 (63.2)
Avoid recreational substances	71 (46.7)
Manage OUD symptoms	25 (16.4)
Live a normal life	14 (9.2)
Improve mental health	11 (7.2)
Employment	8 (5.3)
Maintain or stabilize MAT dose	7 (4.6)
Family-related goals	7 (4.6)
Education	5 (3.3)
Pain management	3 (2.0)
No goal	2 (1.3)
No. of reported goals per patient	
None	2 (1.3)
1	79 (52.0)
2	51 (33.6)
3	15 (9.9)
4	5 (3.3)

Abbreviations: IQR, interquartile range; MAT, medication-assisted treatment; OUD, opioid use disorder.

^a^ Abstinence determined by urine drug screening test result.

^b^ Data available for 145 participants.

^c^ Based on urine drug screens during respective period of interest.

^d^ Percentages sum to greater than 100% because patients were allowed to report multiple treatment goals.

### Patient-Reported Treatment Goals

Altogether, 150 patients (98.7%) reported at least 1 treatment goal, and only 2 (1.3%) reported no goal (Table 1). Participants reported the following goals: taper dose or stop MAT (96 [63.2%]), avoid recreational substances (71 [46.7%]), manage OUD symptoms (25 [16.4%]), and live a normal life (14 [9.2%]). Overall, 26 of 46 participants (56.5%) taking buprenorphine reported a goal of wanting to taper the dose of or stop MAT compared with 70 of 106 participants taking methadone (66.0%); this difference was not significant (χ^2^_1_ = 1.25; *P* = .26). Other PRGs are summarized in Table 1.

We examined goal-specific outcomes for individuals who reported PRGs compared with those who did not report them. Overall, there were no significant differences in goal attainment for any of the other PRGs examined: taper dose or stop MAT (OR, 1.98; 95% CI, 0.88-4.46; *P* = .10), avoid recreational substances (OR, 1.34; 95% CI, 0.65-2.74; *P* = .43), manage OUD symptoms (β coefficient, –0.93; 95% CI, –4.24 to 2.38; *P* = .58), and improve mental health (β coefficient, –0.76; 95% CI, –6.31 to 4.78; *P* = .79) (Table 2).

**Table 2.** Univariable Regression of Patient-Reported Goals and Their Respective Outcome Measures (N = 152)

Goal	OR (95% CI)	β Coefficient (95% CI)	*P* value
Taper dose or stop MAT^a^^,^^b^	1.98 (0.88 to 4.46)	NA	.10
Avoid recreational substances^c^^,^^d^	1.34 (0.65 to 2.74)	NA	.43
Employment^e^	4.33 (0.99 to 18.98)	NA	.05
Manage OUD symptoms^f^	NA	–0.93 (–4.24 to 2.38)	.58
Improve mental health^g^	NA	–0.76 (–6.31 to 4.78)	.79

Abbreviatons: MAT, medication-assisted treatment; NA, not applicable; OR, odds ratio; OUD, opioid use disorder.

^a^ Data available for 150 participants.

^b^ Proportion of individuals with a decreased MAT dose during 3-month study period.

^c^ Data available for 145 participants.

^d^ Proportion of individuals with negative urine drug screening test results for all recreational substances during 3-month study period.

^e^ Proportion of employed participants at study enrollment.

^f^ Total Maudsley Addiction Profile physical score at enrollment.

^g^ Total Maudsley Addiction Profile psychological score at enrollment.

We examined the factors associated with attainment of the 2 most commonly reported goals: taper dose or stop MAT and avoid recreational substances. Overall, 39 of 150 participants (26.0%) had a decreased MAT dose at 3 months. Participants taking methadone had significantly higher odds of having a decreased MAT dose at 3 months compared with those taking buprenorphine (OR, 4.42; 95% CI, 1.40-14.0; *P* = .01; Table 3). No other factor was associated with a decreased MAT dose at 3 months, including the goal to taper dose or stop MAT (OR, 1.90; 95% CI, 0.78-4.63; *P* = .16; Table 3).

**Table 3.** Multivariable Logistic Regression of Patient Covariates and Decreased MAT Dose at 3 Months (N = 150)

Covariate	OR (95% CI)	*P* value
Age in years	1.14 (0.89-1.45)	.30
Female vs male sex	2.18 (0.99-4.79)	.05
Methadone vs buprenorphine treatment	4.42 (1.40-14.0)	.01
MAT dose at study enrollment	0.99 (0.97-1.00)	.09
Length of treatment in years	1.04 (0.81-1.33)	.76
Goal: taper dose or stop MAT	1.90 (0.78-4.63)	.16

Abbreviations: MAT, medication-assisted treatment; OR, odds ratio.

At baseline, 17.1% (n = 26) of our sample had negative UDS results for all recreational substances compared with 29.7% (n = 43 of 145) at 3 months (Table 1). However, none of the factors included in our model were associated with the avoidance of recreational substances at 3 months, including the goal to avoid recreational substances (OR, 1.27; 95% CI, 0.60-2.67; *P* = .53) (Table 4).

**Table 4.** Multivariable Logistic Regression of Patient Covariates and Avoidance of Recreational Substances at 3 Months (N = 145)

Covariate	OR (95% CI)	*P* value
Age in years	1.08 (0.87-1.33)	.49
Female vs male sex	0.59 (0.28-1.23)	.16
Methadone vs buprenorphine treatment	1.11 (0.39-3.19)	.85
MAT dose at enrollment	0.99 (0.98-1.01)	.44
Length of treatment in years	0.92 (0.74-1.16)	.50
Goal: avoid recreational substances	1.27 (0.60-2.67)	.53

Abbreviations: MAT, medication-assisted treatment; OR, odds ratio.

## Discussion

We identified 10 unique PRGs in this cohort of youths receiving MAT for OUD; most of these goals were often not examined in the existing literature.^16,17,18,19,20,21,22^ For all PRGs examined, participants who reported the goal did not have a significantly higher odds of achieving it compared with those who did not have that PRG. Furthermore, multivariable analyses of factors associated with a decreased MAT dose and avoidance of recreational substances did not show an association between reporting these respective goals and attaining them. Although identification of PRGs is a crucial aspect of patient-centered care,^1^ our data suggest that youths’ goals when receiving MAT for OUD are not being specifically attained. Therefore, we have identified a need to better incorporate these goals into clinical care.

The most commonly reported goal was to taper the dose or stop MAT. Although 26.0% of participants successfully decreased their MAT dose during the 3-month study period, reporting this goal was not associated with increased odds of attaining it. However, receiving methadone compared with buprenorphine was associated with increased odds of decreasing the MAT dose. Conversely, individuals receiving methadone were not significantly more likely to report the goal to taper dose or stop MAT compared with individuals receiving buprenorphine. Therefore, neither the difference in adverse effect profile, toxic effects, and tolerability between these 2 medications nor the stigma associated with methadone^36^ fully explains youths’ motivations for wanting to taper or stop MAT. Given the risk of adverse outcomes demonstrated with the use of methadone in the adult population,^37,38,39,40,41^ this result may reflect clinicians’ concerns about the use of methadone for youths rather than patient preferences. Although this study does not explore the reasons why youths would want to taper or stop MAT, clinicians may wish to explore these questions in greater detail to ensure that patients’ goals are understood.

Because clinicians are hesitant to prescribe MAT for youths,^41^ there is no guidance on safely discontinuing MAT in this age group, to our knowledge.^37^ A double-blind, placebo-controlled, multicenter randomized clinical trial found that a 56-day buprenorphine taper led to increased abstinence and treatment retention compared with a 28-day taper.^20^ However, guidelines do not suggest when it is appropriate to consider tapering the dose of MAT for youths.^37^ Without robust, age-specific, long-term follow-up data, it is unclear whether the same concerns from the adult literature^37,38,39,40,41^ apply to youths. Given that most of our cohort wanted to taper their dose of MAT or stop MAT altogether, future research should focus on developing safe, effective, and tolerable tapering strategies for this population.

### Strengths and Limitations

This study has some strengths. We found that enrollment in MAT was associated with a decrease in the proportion of participants with positive UDS results for recreational substances. However, patients who reported wanting to avoid recreational substances did not have higher odds of achieving this outcome. Furthermore, the use of UDS results as an objective measure of abstinence is a strength of this study. This finding further reinforces the conclusion that patients’ goals were not being specifically elicited, addressed, or attained in treatment. One limitation to this conclusion is that this study did not explore the relative importance of the PRGs. However, because most patients (130 [85.5%]) reported only 1 or 2 goals, we do not think that the reporting of a low-priority goal is a likely explanation for this finding. This lack of specific goal attainment places an even greater emphasis on the need to actively incorporate patients’ goals into their treatment plan.

To our knowledge, this is the first study of PRGs among youths with OUD. To address potential sources of bias, we used a pragmatic study design with minimal exclusion criteria. To reduce selection bias, we recruited participants in clinical settings in which they routinely receive care; all eligible participants were approached. To minimize response bias, interviews were conducted by research personnel who were not involved in patient care. We also obtained data from clinical records to supplement participant interviews. In addition, the use of the open-ended interview question “What are your goals in treatment?” is a strength of this study because it allowed participants to voice what they felt was important to them, rather than selecting from a predefined list. Statistically, regression models displayed good fit, lack of multicollinearity, and no extreme outliers.

This study also has some limitations. Our data are susceptible to healthy user bias, volunteer bias, and social desirability bias.^42,43^ Because of our observational design, we could not control for unknown or unmeasured factors associated with our outcomes of interest. In addition, the number of individuals who reported the goals of employment and improving mental health was relatively low to draw definitive conclusions about our treatment program’s ability to help participants achieve these specific goals. Furthermore, we collected the MAP questionnaire and employment data at study enrollment rather than at the 3-month follow-up period. This measurement timeline assumes that participants who reported the goals of employment, managing OUD symptoms, and improving mental health also had these goals in the 12 months before study enrollment. Moreover, the MAP questionnaire was not designed to assess specific OUD-related symptoms.^32^ However, it was intended for assessing the treatment programs’ outcomes in substance use disorders.^31^ For the remaining goals, it is possible that a 3-month follow-up period was not long enough to allow some participants to achieve their stated goals. However, these limitations do not eliminate the need for the consideration and evaluation of PRGs in clinical practice, if we are to strive for patient-centered care.

## Conclusions

To our knowledge, this is the first study to outline PRGs for youths receiving MAT for OUD. Previous studies use abstinence and treatment retention as measures of successful treatment. However, our results demonstrated that patients often have highly variable treatment goals that are not captured by these measures.^26^ Moreover, individuals who reported certain goals had similar odds of achieving them as those who did not report those goals, highlighting the need to specifically inquire about and monitor PRGs. To ensure that patients can achieve their goals, we recommend that MAT research and treatment programs regularly inquire about PRGs and use robust outcome measures, such as the MAP, to monitor their success. Of particular interest is the need to develop age-specific guidelines for safely tapering off the dose of or stopping MAT for OUD among youths. Because a diagnosis of OUD has significant implications for the psychosocial trajectory of this population, it is necessary to ensure that both clinicians and researchers can accurately track these broad dimensions of care.

## Notes

Supplement. **eTable 1.** Summary of Themes Derived From Qualitative Analysis (n = 152) **eTable 2.** Demographic and Clinical Characteristics (n = 152)
