# Supplementary material for: Patient-Reported Goals of Youths in Canada Receiving Medication-Assisted Treatment for Opioid Use Disorder
Source: JAMA Netw Open. 2021 Aug 5;4(8):e2119600. doi: 10.1001/jamanetworkopen.2021.19600 (PMC8343465; doi:10.1001/jamanetworkopen.2021.19600)
Supplement: Supplement. — eTable 1. Summary of Themes Derived From Qualitative Analysis (n = 152) eTable 2. Demographic and Clinical Characteristics (n = 152) [file jamanetwopen-e2119600-s001.pdf]

Supplementary Online Content

Chai D, Rosic T, Panesar B, et al. Patient-reported goals of youths in Canada receiving medication-assisted treatment for opioid use disorder. *JAMA Netw Open.* 2021;4(8):e2119600. doi:10.1001/jamanetworkopen.2021.19600

eTable 1. Summary of Themes Derived From Qualitative Analysis (n = 152)
eTable 2. Demographic and Clinical Characteristics (n = 152)

This supplementary material has been provided by the authors to give readers additional information about their work.

© 2021 Chai D et al. *JAMA Network Open.*

eTable 1. Summary of Themes Derived From Qualitative Analysis (n = 152)

Themes	Node	Description
"Taper dose or stop MAT[a]"	Stop treatment	Comments relating to getting off MAT [a] treatment, whether suboxone or methadone, regardless of the urgency or timeframe stated.
	Reduce dose	Comments relating to coming off MAT [a] slowly, regardless of whether they mention stopping treatment or not; comments coded here focus on reducing dose and do not use taper.
	Taper dose	Comments relating to coming off MAT [a] slowly, regardless of whether they mention stopping treatment or not.
"Avoid recreational substances"	Sobriety	Comments relating to getting clean, staying clean, achieving abstinence, maintaining abstinence and stopping drug related dependency (on any drug).
	Limit negative influences	Comments relating to staying away from drug inducing people and achieving a drug free environment.
"Manage OUD [b] symptoms"	Control cravings	Comments relating to the prevention of cravings, whether to MAT [a] programs or illicit drugs.
	Avoid withdrawal	Comments relating to the prevention of side effects from drugs, including MAT [a] programs.
"Live a normal life"	Establish a stable life	Comments relating to living a life that is more stable than what they are living currently and living a life that has less stressors; Establishment of a healthier lifestyle than what they currently have.
"Improve mental health"	Manage mental health	Comments relating to mental illness or the establishment of mental wellbeing by outlining general or specific symptoms such as maladaptive moods and lack of motivation.
	Management of stressors	Comments relating to the seeking of mental health treatment either through the clinic or outside of the clinic to manage stressors in a healthy manner.

© 2021 Chai D et al. *JAMA Network Open.*

Employment	Establishment of personal financial stability	Comments relating to a change of career, getting a job, fulfilment of financial needs.
"Maintain or stabilize MAT [a] dose"	Stabilize dose	Comments relating to the stabilization of the current dose, optimization of dose, or maintenance of dose, due to benefits of MAT [a].
Family-related goals	Establishment of familial stability	Comments relating to the fulfilment of financial, practical, and emotional needs of family members.
	Regaining family members	Comments related to the re-establishment of familial connection (physical or emotional).
Education	Restarting education	Comments relating to apprenticeship, trades and the notion of going back to school.
Pain management	Pain	Comments relating to the use of MAT [a] to manage pain/chronic pain and general comments on pain management as a goal.
No goal	No change in treatment	Comments relating to having no goals or being uncertain of goals they may have.

[a] Medication-assisted treatment
[b] Opioid use disorder

© 2021 Chai D et al. *JAMA Network Open.*

eTable 2. Demographic and Clinical Characteristics (n = 152)

**Characteristic**	**Participants with complete data (n = 145)**	**Participants missing 3-month UDS [a] data (n = 7)**	***p-value***
Age, years; mean (SD) [range]	22.8 (1.8) [16.7 - 24.9]	22 (2.2) [18 -23.7]	.26
Sex, number of females; n (%)	66 (45.5%)	4 (57.1%)	.36
**Socioeconomic Factors**			
Patients with children; n (%)	51 (35.2%)	5 (71.4%)	.05
Currently employed; n (%)	8 (5.5%)	0 (0%)	.52
**Level of Education**			
Less than high school; n (%)	63 (43.5%)	7 (100%)	*.003
High school completed; n (%)	73 (50.3%)	0 (0%)	*.009
Post-secondary education in progress or completed; n (%)	9 (6.2%)	0 (0%)	.50
**MAT [b] History**			
Type of MAT [b] ; n (%)			
Methadone	102 (70.3%)	4 (57.1%)	.46
Buprenorphine	43 (29.7%)	3 (42.9%)	.46
Baseline dose in mg/day; mean (SD)			
Methadone	60.0 (33.2)	4 (10.9)	<.001
Buprenorphine	11.0 (6.8)	3 (6.4)	.009
Length of treatment in years; median (IQR)	0.92 (1.75)	0.17 (0.39)	.09
**Substance Use History [c]**			
Number of participants who were opioid abstinent; n (%)			
Baseline	49 (33.8%)	3 (42.9%)	.62
3 months	78 (53.8%)	-	
Any recreational substance use; n (%)			
Baseline	121 (83.5%)	5 (71.4%)	.41
3 months	102 (70.3%)	-	
Cocaine non-user [d]; n (%)			
Baseline	58 (40%)	4 (57.1%)	.37
3 months	76 (52.4%)	-	
Amphetamine non-user [d]; n (%)			
Baseline	88 (60.7%)	6 (85.7%)	.18
3 months	102 (70.3%)	-	

© 2021 Chai D et al. *JAMA Network Open.*

Methamphetamine non-user [d]; n (%)			
Baseline	106 (73.1%)	6 (85.7%)	.46
3 months	115 (79.3%)	-	

SD = Standard deviation, IQR = Interquartile range
[a] Urine drug screen
[b] Medication-assisted treatment
[c] Abstinence determined by urine drug screen
[d] Based on urine drug screens during respective period of interest

© 2021 Chai D et al. *JAMA Network Open.*
